# Evaluating Transperineal Prostate Biopsies Performed by a New Operator: A Prospective Clinical Audit at Dorset County Hospital

**DOI:** 10.7759/cureus.54951

**Published:** 2024-02-26

**Authors:** Muhammad Arshad Irshad Khalil, Naveed Afzal, Muhammad S Bajwa

**Affiliations:** 1 Department of Urology, Dorset County Hospital NHS Foundation Trust, Dorchester, GBR

**Keywords:** prostate biopsy, multiparametric prostate mri, gleason grade, pirads score, trus, transperineal biopsy

## Abstract

Introduction

The transperineal (TP) approach for prostate biopsy offers advantages such as a low risk of infection, the ability to target lesions in difficult locations, and a rapid acquisition of proficiency. This prospective clinical audit aims to evaluate the outcomes and patient experiences of TP prostate biopsies performed by a new operator to determine the feasibility of adopting the TP biopsy as the primary method for prostate evaluations.

Methods

The study included all patients who underwent a TP prostate biopsy from August 1 to September 30, 2022, at Dorset County Hospital National Health Service Foundation Trust. The operator, a member of the urology team, had recently begun performing these biopsies independently after completing a four-month supervised training program and receiving approval from two consulting trainers. The biopsy technique was evaluated based on diagnostic yield and patient experience, comparing pre-procedure imaging results with histology reports and analyzing patient-completed questionnaires.

Results

Among the 42 patients, the cancer detection rate was 79%. The highest core positivity rate was 100% in two patients (5%), with 90% in 11 patients (26%). Of the patients, 57% showed complete agreement between magnetic resonance imaging findings and histology. A questionnaire assessing patient experience received a 64% response rate. The most common pain score reported was 2 (on a scale of 0-10), noted in 25% of patients. Most reported mild lower urinary tract symptoms (88%) and mild hematuria (85%). Of the patients, 44% rated their overall satisfaction as 10 (on a scale of 0-10), and no urinary tract infections were reported.

Conclusion

The findings support the adoption of TP biopsy as the primary method for prostate biopsies due to its short learning curve, high diagnostic yield, and favorable patient satisfaction. Training for new operators should be encouraged to achieve this goal.

## Introduction

Prostate cancer is the second most common cancer in men and the fifth leading cause of death worldwide [1]. Prostate biopsy plays a crucial role in the diagnosis and management of prostate cancer. Biopsy techniques have significantly evolved over the past several decades [2-4]. The transrectal (TR) approach, utilizing a transrectal ultrasound (TRUS) probe, remains the most widely used biopsy technique globally [5] due to its simplicity, low cost, and feasibility under local anesthesia. However, this method carries notable disadvantages, including a higher risk of infection and the potential to miss cancerous lesions in areas that are more challenging to access within the prostate [6]. An alternative, the transperineal (TP) approach, avoids passing through the rectum, thereby reducing the risk of infection. It also enables practitioners to target areas that are difficult to access with the TR approach, for example, apical and anterior zones. Despite logistical challenges, the European Association of Urology now recommends replacing the TR approach with the TP approach [7].

This prospective clinical audit of patients undergoing TP prostate biopsies over two months involves a single member of the urology team at Dorset County Hospital National Health Service Foundation Trust (DCHFT), who has begun practicing independently after completing a four-month supervised training period and receiving approval from two consultant trainers. The training was structured into phases of observation and assistance during the first month, followed by performing under direct supervision in the second and third months, and ended with indirect supervision during the last month. On average, the trainee was given exposure to eight procedures per week throughout the training period. Our audit aims to evaluate the diagnostic yield and patient experiences to assess whether the TP approach can replace the TR approach as the preferred method for prostate biopsy. Additionally, this audit seeks to encourage the training of new operators and to expand the service provision.

## Materials and methods

Prior to commencement, we obtained approval from the hospital audit committee and registered the study (Approval No.: 5634). Our research design was a prospective case series, including all patients undergoing TP prostate biopsy under general anesthesia with template mapping by the specified operator from August 1 to September 30, 2022, selected via convenience sampling. We evaluated the outcomes based on diagnostic yield and patient experiences.

Diagnostic yield was measured using variables such as prostate size, Prostate Imaging Reporting and Data System (PIRADS) scores from multiparametric magnetic resonance imaging (mpMRI) scans (further broken down by scores from the right and left peripheral zones (PZ), transitional zone (TZ), and apical zone), overall cancer detection rate, total number of biopsy cores per patient, percentage of cancerous cores, and Gleason score categorization of detected cancers.

To assess the correlation between mpMRI scans and histological findings, we utilized the “Dorchester map,” a collaborative development between the urology and histopathology departments at DCHFT. Patient experience was gauged through a questionnaire distributed via email, covering pain levels (on a 0-10 scale), post-procedure lower urinary tract symptoms (LUTS; rated from mild to severe, with mild being self-resolving and severe requiring treatment), hematuria severity (from mild to severe, with mild being intermittent and resolving quickly and severe requiring medical intervention), instances of urinary tract infections (UTIs) necessitating antibiotics, and overall satisfaction (on a 0-10 scale). The collected data were analyzed and presented graphically.

## Results

During the study period, 42 patients underwent TP prostate biopsies. The prostate-specific antigen levels ranged from a minimum of 0.59 ng/ml to a maximum of 791 ng/ml. Prostate sizes varied from 17 cc to 124 cc. The most frequent PIRADS score observed on mpMRI scans was 4, identified in the PZ of 27 patients, followed by a score of 5 in 18 patients. Notably, two patients exhibited a PIRADS 4 lesion within the TZ.

Diagnostic yield

**Figure 1:**
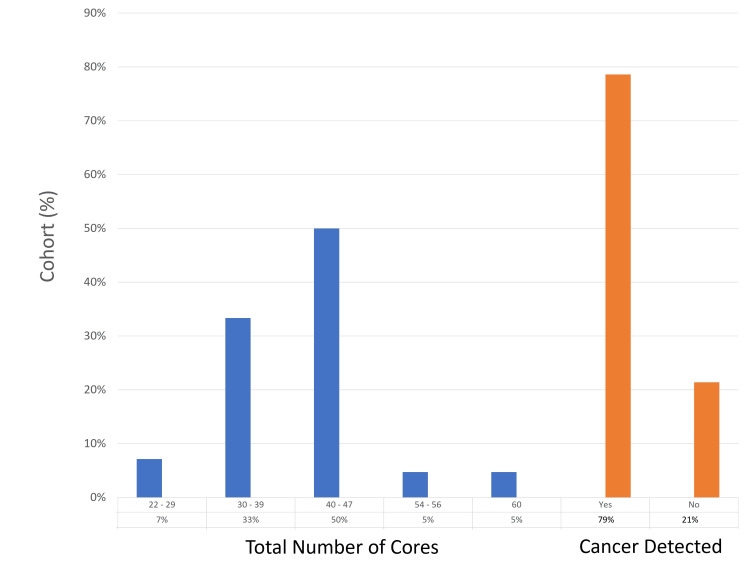
Number of cores per patient and overall cancer diagnosed

**Figure 2:**
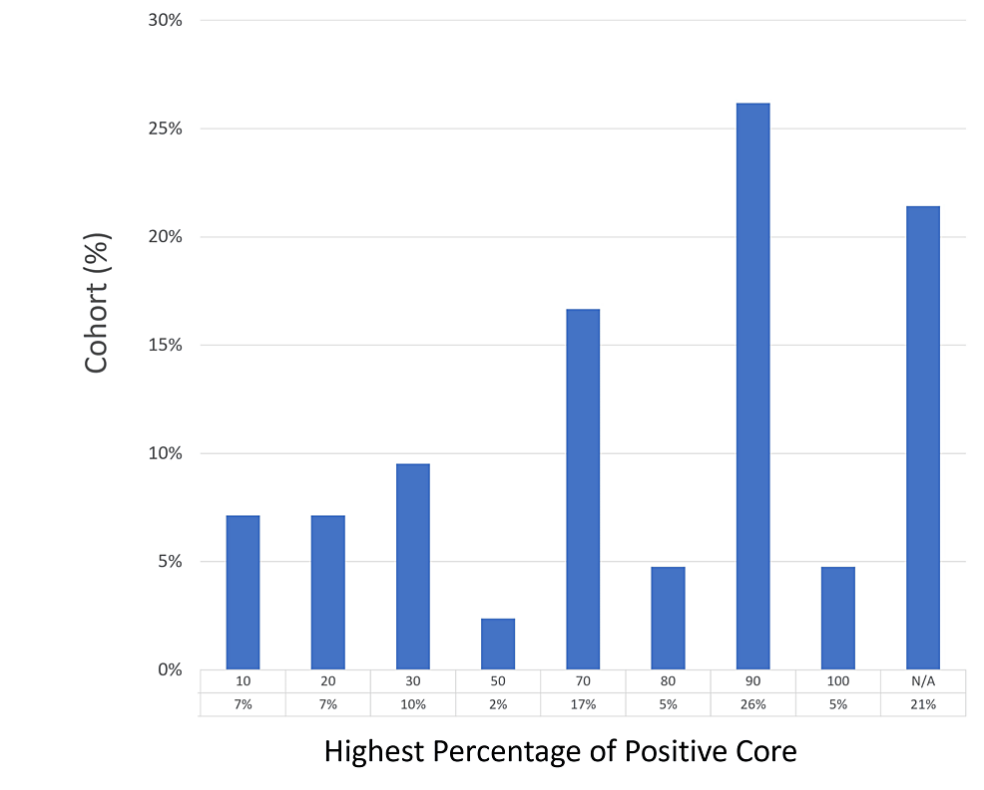
Individual core quality by the percentage of cancers detected N/A, not applicable.

**Figure 3:**
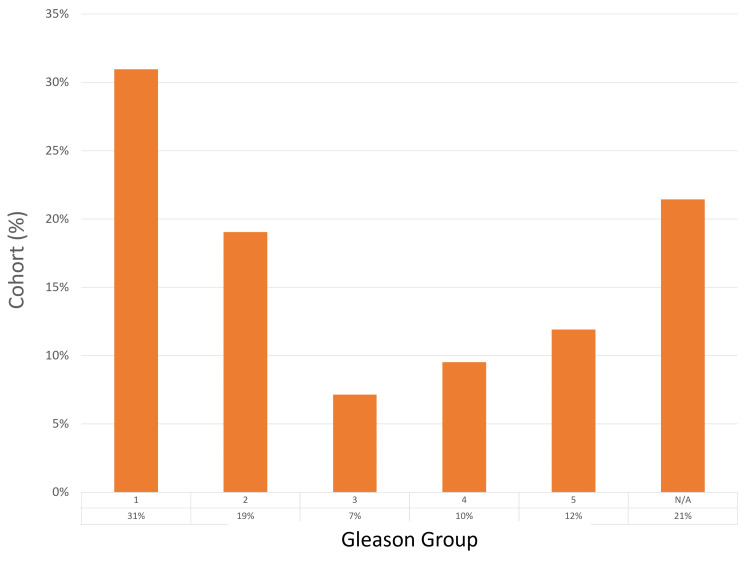
Gleason grade group analysis in positive cores N/A, not applicable.

The detection rate of prostate cancer in this cohort was 79% (33 of 42 patients), with the highest number of biopsy cores taken from a single patient being 60. Two patients (5%) had all their sampled cores testing positive for cancer. The most common Gleason score was grade group 1, found in 13 (31%) patients, with grade group 2 observed in eight (19%) patients. A complete agreement between mpMRI findings and histological results was achieved in 24 (57%) patients, while a partial agreement was noted in six (14%). The diagnostic yield results are detailed in Figures 1-3.

**Figure 4:**
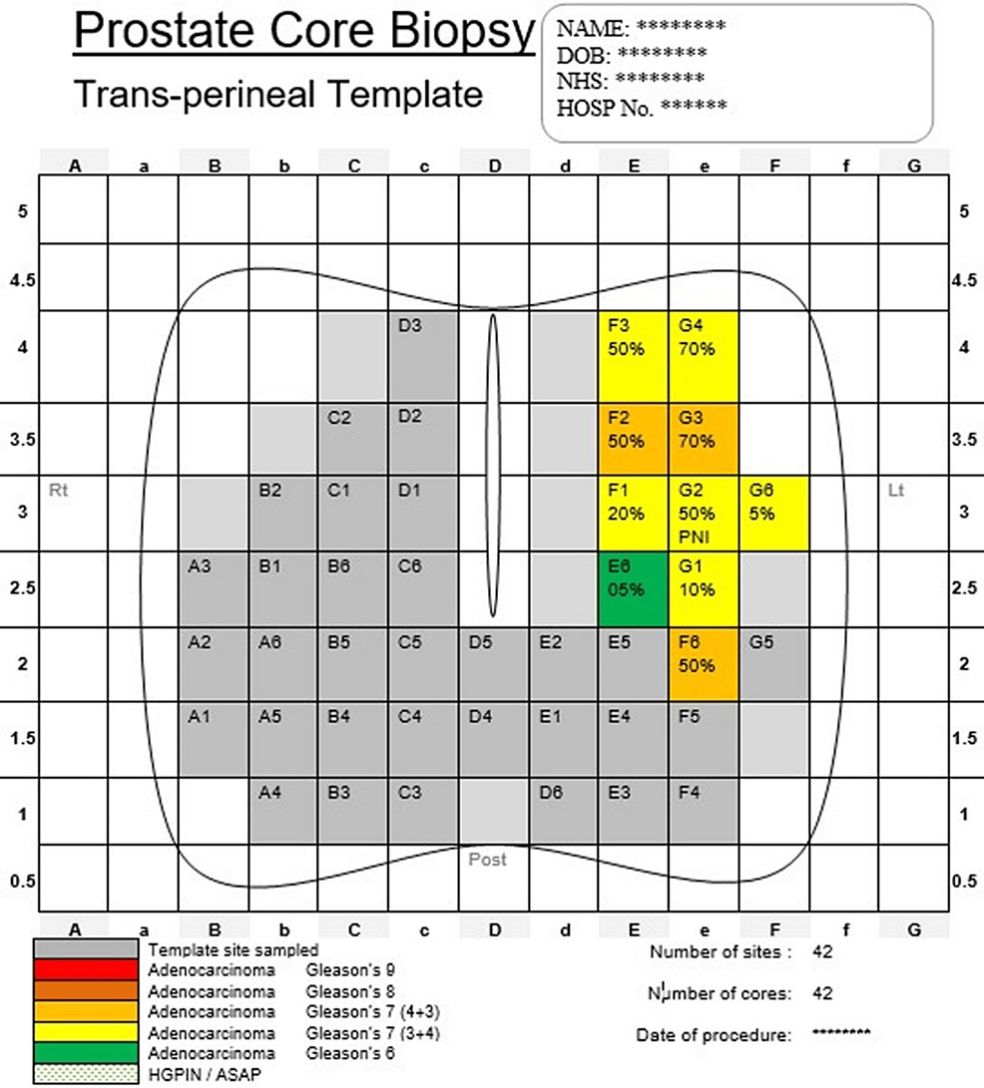
Dorchester map showing cancer detected in the left transitional zone DOB, date of birth; NHS, National Health Service; HOSP, hospital; PNI, perineural invasion; HGPIN, high-grade prostate intraepithelial neoplasia; ASAP, atypical small acinar proliferation.

In this group, 13 patients (31%) had tumors located anteriorly or apically, where the biopsy successfully obtained representative samples and confirmed cancer presence. Intriguingly, in one case, an anterior tumor was detected despite being missed by mpMRI. A representative “Dorchester Map” illustrating cancer in the left anterior TZ is displayed in Figure 4.

Patients' experience

**Figure 5:**
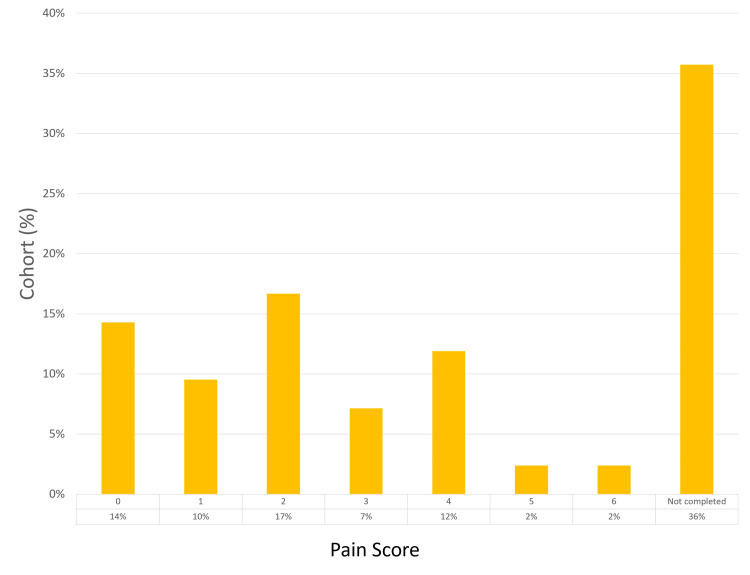
Post-procedure pain score analysis

**Figure 6:**
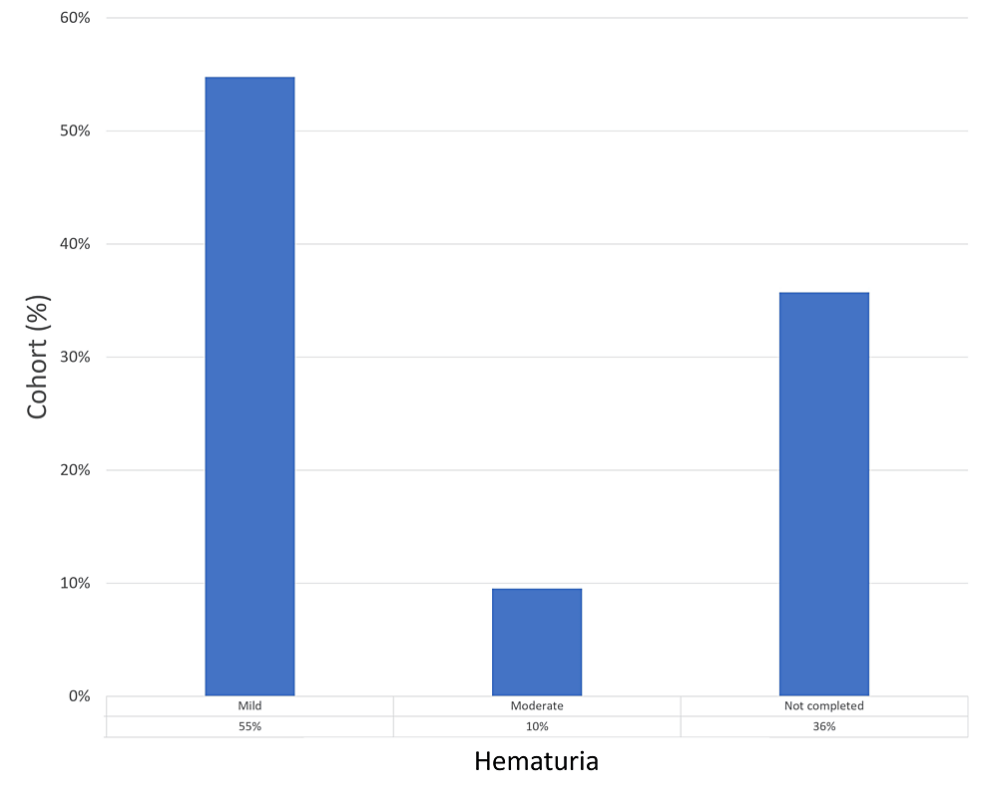
Post-procedure hematuria scores analysis

**Figure 7:**
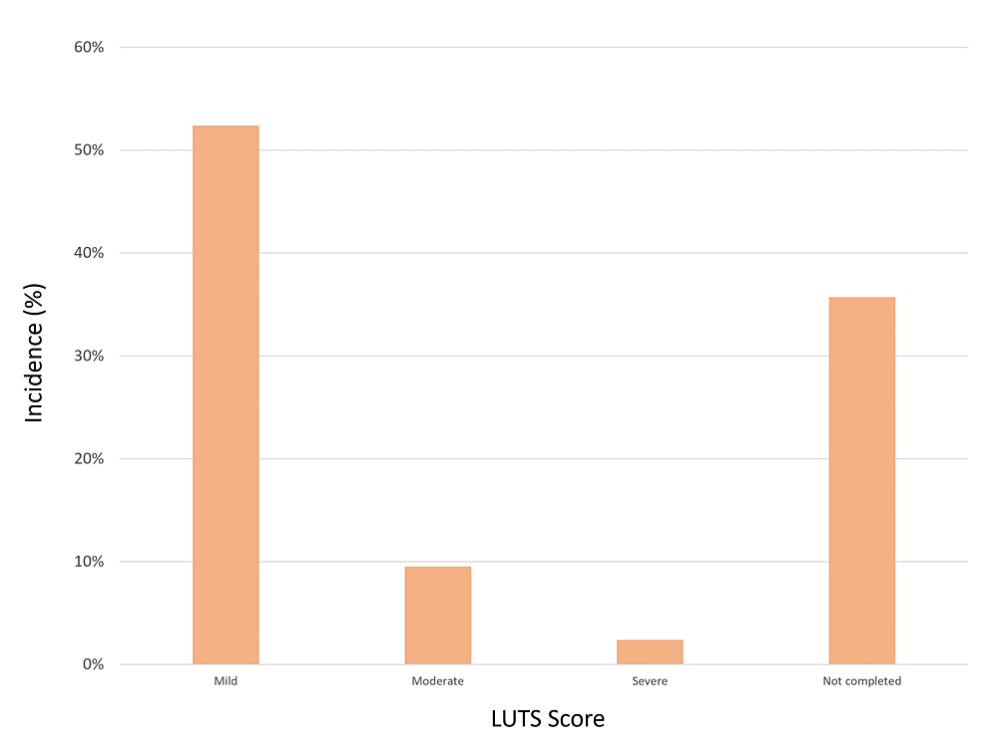
Post-procedure LUTS scores analysis LUTS, lower urinary tract symptoms.

**Figure 8:**
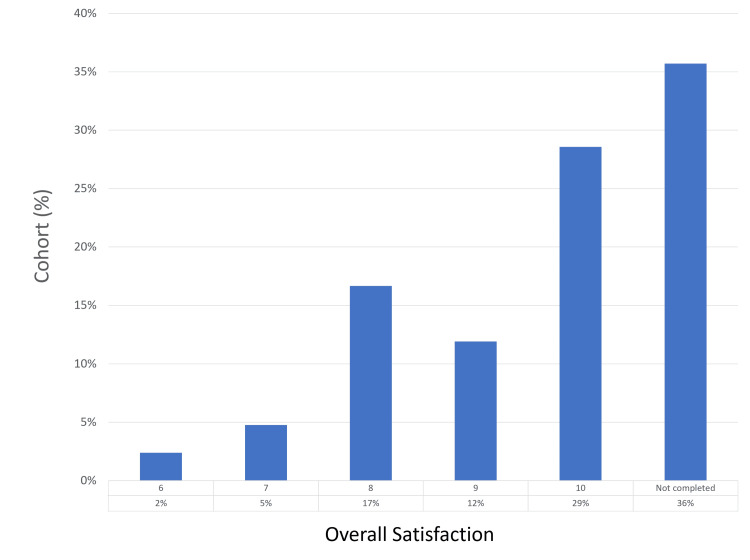
Patient-reported overall satisfaction scores

Of the study participants, 27 (64%) returned completed questionnaires. Regarding pain experienced during the procedure, the most frequent score reported was 2 (by 25% of respondents), with a score of 0 being the next most common (22%). Only one patient (2%) reported a maximum pain score of 6. Mild hematuria was reported in 23 (85%) patients, with moderate hematuria observed in four (15%) patients; none experienced severe hematuria. Regarding LUTS post-procedure, 22 patients (81%) reported mild symptoms, four (15%) reported moderate symptoms, and one (2%) experienced severe LUTS, leading to retention - a pre-existing condition. No patients reported UTIs. In terms of overall satisfaction with the biopsy process and outcomes, scores of 10 were given by 12 (44%) respondents, followed by scores of 8 (25%) and 9 (19%). The patient experience findings are summarized in Figures 5-8.

## Discussion

The TR approach, employing TRUS, continues to be the predominant method for prostate cancer biopsies. However, this technique is not without its inherent risks and limitations. Originally established as the standard practice following pivotal research by Hodge et al., the sextant biopsy method, which takes six cores, was initially deemed sufficient [8]. Over time, adopting a 10-to-12 laterally directed biopsy protocol has become more prevalent, as it is believed to detect 99% of cancers compared to the 72.6% detection rate of the standard sextant protocol. A prospective randomized study by Naughton et al. observed no difference in discomfort between the two methods, though the incidence of hematospermia (89% vs. 71%) and rectal bleeding (24% vs. 10%) increased when 12 biopsies were taken [9].

Since its initial description in 1981, the TP biopsy route under ultrasound guidance has significantly evolved. Its primary advantage lies in bypassing the rectal route, thus reducing the risk of introducing rectal bacteria into the prostate, which could lead to UTIs or sepsis. Moreover, it has demonstrated superior diagnostic outcomes, particularly for lesions in the anterior and apical sections of the prostate, which are challenging to reach via the TR approach [10,11]. The integration of cognitive fusion, MRI-TRUS fusion, and tools such as the template grid and PrecisionPoint™ transperineal access system has made the TP route the preferred method for biopsying lesions difficult to access through the TR approach.

Another significant drawback of the TRUS-guided biopsy is its association with a considerable risk of infection. Despite prophylactic antibiotic use and rectal cleansing, there remains a risk of implanting rectal bacteria into the prostate [12]. Norwegian patient registry data from 2011 to 2017 revealed a 10% hospital admission rate for infections post-TR prostate biopsy [13]. A systematic review and meta-analysis, including seven randomized studies with 1,330 patients, indicated that infectious complications were significantly higher following TR biopsies than TP biopsies [14]. A systematic review of 165 studies involving 162,577 patients also reported sepsis rates of 0.1% for TP biopsies and 0.9% for TR biopsies [15]. A population-based UK study involving 73,630 patients found lower readmission rates for sepsis following TP biopsies compared to TR biopsies (1.0% vs. 1.4%, respectively) [16]. Consequently, the European Association of Urology recommends favoring the TP approach over the TR approach, despite potential logistical hurdles [7].

Our study’s findings suggest that despite the current TP approach requiring general anesthesia (GA), it remains acceptable to most patients, particularly when they are fully informed about the associated risks and can make an informed decision. This proactive communication strategy also helps to alleviate post-procedure concerns and prevents undue panic regarding potential complications.

The limitations of our study include a relatively small sample size, the exclusive use of GA for all procedures, a relatively higher number of cores per prostate, and a lack of comparison with TR prostate biopsies by the same operator. Future research with larger sample sizes, procedures performed under local anesthesia, and a direct comparison with the TR approach could provide further insights into preferences between biopsy approaches. It is anticipated that with further experience, a new operator shall be able to reproduce a similar diagnostic yield with a lesser number of cores, thus further improving patient satisfaction with the TP approach.

## Conclusions

The TP biopsy technique should be adopted as the preferred method for diagnosing prostate cancer. It offers a short learning curve, high diagnostic yield, and the ability to target lesions in difficult-to-reach areas. Moreover, it is associated with acceptable morbidity and high patient satisfaction, even when performed by operators with limited experience.
